# VTA-forebrain connectivity moderates adaptive behavior

**DOI:** 10.1007/s00429-026-03071-2

**Published:** 2026-02-03

**Authors:** Sadia Islam Sinza, Kwon Choi, Ignitius Ezekiel Lim, Madison Ashley Williams, Olalekan Michael Ogundele

**Affiliations:** Department of Comparative Biomedical Sciences, Louisiana State University School of Veterinary Medicine, Baton Rouge, LA USA

**Keywords:** Ventral tegmental area, Hippocampus, Prefrontal cortex, Valence, Novelty, Adaptive behavior

## Abstract

Neural representation of the environment is pertinent for adaptive behaviors. Such neural maps are processed in the hippocampus and contain information about spatial locations, novelties, context, and cues for context discrimination. In conjunction with other subcortical structures, a key function of the hippocampus is to compare newly detected novelties derived from the environment with previously stored cortical memories. These neural events underscore the role of the hippocampus as a “comparator” and “hub” for sorting novelties to determine priority and weight assignments for long-term cortical storage. To propagate these cognitive processes, mesocorticolimbic ventral tegmental area (VTA) inputs to the hippocampus and prefrontal cortex (PFC) compute detected novelties to derive frameworks for context discrimination, valence designation, and experience-based learning. Additionally, reciprocal connections between the VTA-hippocampus and VTA-PFC circuits are engaged for guided decisions and expression of adaptive behaviors. The significance of the functionally linked VTA-hippocampus and VTA-PFC axis in learning and guided decision-making has been generally described within the GO-STOP framework, which involves the coordinated activation or inhibition of ensembles in the prelimbic (PrL) and infralimbic (IL) cortices, among others. Although the GO-STOP hypothesis has been excellently described in recent works, this review evaluates the functional continuum between the VTA-hippocampus loop and VTA regulation of cortical-linked executive cognitive processes which underscore the expression of adaptive behaviors, with associated implications in psychological and neurodevelopmental disorders.

## Introduction

The brain neurally encodes internal and external states. Detection of these states as sensory, spatial, and contextual events guides future decisions through learned experience. Thus, cognitive processes and neural circuits that propagate learning, memory, valence, and novelty are pertinent for adaptive behaviors (Braun et al. 2018; Capuzzo and Floresco 2020; Cowan et al. 2021; Han et al. 2020; Hardung et al. 2021; Herry and Johansen 2014; Mair et al. 2021). The gateway to these processes is “hippocampal connectivity”, which compares pre-existing and newly formed memories (Castillo Diaz et al. 2022; Cowan et al. 2021; Duszkiewicz et al. 2019; Ghanbarian and Motamedi 2013; Han et al. 2020; Lisman and Grace 2005; Ntamati and Luscher 2016; Ripolles et al. 2016; Rossato et al. 2009). However, determining the valence of such novelties occurs in conjunction with the mesocorticolimbic, lateral habenula, amygdala-hypothalamus axis, and other related circuits. Therefore, learned experiences are derived through computation across ensembles in these brain regions and are reapplied to guide future decisions about space, sensory cues, or contexts (Keinath et al. 2014; Kim et al. 2016; Li et al. 2019; Michel et al. 2024; Miller et al. 2001; Paquelet et al. 2022; Redondo et al. 2014; Wu et al. 2022; Yizhar and Klavir 2018; Zelikowsky et al. 2014; Zhang and Li 2018).

The hippocampus and prefrontal cortex (PFC) are functionally related and anatomically linked (Abbas et al. 2018; Bowman and Dennis 2016; Euston et al. 2012; Godsil et al. 2013; Herweg et al. 2016; Johnson et al. 2021; Li et al. 2015; Mair et al. 2021; O'Neill et al. 2013; Park et al. 2021; Sotres-Bayon et al. 2012; Takita et al. 1999; van Kesteren et al. 2012; Wang et al. 2021; Zelikowsky et al. 2014; Zielinski et al. 2019). Circuit tracing studies have demonstrated robust connections between the hippocampus and PFC and other indirect connections through the reuniens thalamic nucleus (Messanvi et al. 2023; Prasad and Chudasama 2013; Vertes 2006). The hippocampus—in tandem with the prefrontal cortex—is pertinent for the formation of working memory for instantaneous recollections (Baddeley et al. 2011; Funahashi 2017; Giovannini et al. 2017; Johnson et al. 2021; O'Neill et al. 2013; Sasaki et al. 2018; Spellman et al. 2015; Woldeit and Korz 2010). Therefore, spatial, social, and sensory cues are processed here, and their lesions have been established to cause anterograde and partial retrograde amnesias (Knierim 2015; Anand and Dhikav 2012). On the same note, ablation of the prefrontal/perirhinal cortex and its associated anteromedial thalamic regions cause various forms of anterograde and retrograde amnesias (Kapur 1993; Markowitsch et al. 1993; Sigwald et al. 2020; Winocur and Moscovitch 1999). Other cortical and subcortical regions are also central to the neural framework of learning and cognitive functions. In addition to the forebrain centers, brain regions associated with adaptive behaviors, especially the ventral tegmental area (VTA) (Lisman and Grace 2005; Mair et al. 2021; Ripolles et al. 2016; Rossato et al. 2009; Sasaki et al. 2018), accumbens, amygdala, lateral habenula, brainstem raphe nuclei, and hypothalamus—guide memory processing and their relevance for long-term cortical storage.

The focus on the mesocorticolimbic system and ventral tegmental projections to the hippocampus and prefrontal cortex is well justified because of the diversified cell populations, the reach of presynaptic axons of these cells, and their role in the neural computation and predictions of adaptive-linked events (Adell and Artigas 2004; Bouarab et al. 2019; Cowan et al. 2021; Geisler et al. 2007; Hu 2016; Lammel et al. 2014; Lisman and Grace 2005; Montardy et al. 2019; Ntamati and Luscher 2016; Perez-Lopez et al. 2018; Yoo et al. 2016; Zell et al. 2020). The cell populations present in the VTA include dopamine, GABA, and glutamate-releasing neurons. In addition, some neurons that co-release at least two of these neurotransmitters have been identified. Anatomically, dopamine-releasing neurons are abundant in the VTA, forming around 65% of the total cell population. The VTA is also rich in gamma-aminobutyric acid (GABA)-producing neurons and a modest population of glutamate neurons in the medial VTA (Bouarab et al. 2019; Cai and Tong 2022; Adell and Artigas 2004; Ntamati and Luscher 2016). Chemical neuroanatomy of the VTA, and more recently, genetic characterization of mRNAs via high-throughput gene sequencing and in situ hybridization, confirmed populations of VTA cells co-release neurotransmitters. Populations of VTA dopamine neurons co-release glutamate and GABA, and some glutamate neurons co-release dopamine (Cai and Tong 2022; Sanchez-Catalan et al. 2014; Faget et al. 2016; Stetsenko and Koos 2023; Soden et al. 2020). Co-transmission and co-release from VTA neurons represent an additional layer of synaptic modulation within associated neural circuits where these neurotransmitters systems converge, directly influencing spike timing–dependent plasticity (STDP)— a fundamental learning mechanism that depends on the precise timing of pre- and postsynaptic spikes. For example, dopaminergic input to hippocampal CA1 has been shown to regulate STDP by enabling the conversion of LTD into LTP under specific timing conditions. Additionally, projections from VTA GABAergic and glutamatergic neurons to the dentate gyrus (DG) provide co-released inputs that inhibit DG neurons, potentially shaping memory-related circuit dynamics. Thus, VTA-originating co-transmission introduces a critical source of variability and plasticity within hippocampal circuits, shaping how information is integrated and stored (Brzosko et al. 2015; Ntamati and Luscher 2016). This increases the functional complexity of neural circuits involving VTA projections that innervate forebrain centers in two main ways. First, target brain regions, including the hippocampus and cortex, are innervated by a combination of glutamate, GABA, and dopamine-releasing neurons (Taylor et al. 2014; Tang et al. 2020; Gorelova et al. 2012; Carr and Sesack 2000). The anatomical proportionality of these terminals mostly conforms with the neuronal and chemical cytoarchitecture of the targets, such that the accumbens receive larger dopamine inputs than the cortex, and the hippocampus has robust glutamate VTA inputs compared to the dorsal raphe nucleus (DRN) (Lammel et al. 2014; Barbano et al. 2024; Beier et al. 2015; Nguyen et al. 2021; Mingote et al. 2019). Secondly, there are persistent experimental constraints—through current experimental modulation methods—to fully dissect the interdependencies of these neurotransmitters or determine the gradation for their release at target sites. Dopamine originating from the VTA is a key regulator of hippocampal plasticity. Dopaminergic input from the VTA and substantia nigra pars compacta (SNc) primarily acts through D1 receptors in the dorsal hippocampus, facilitating the encoding of contextual and spatial information and supporting LTP induction. Although D1 receptors are most strongly associated with these plasticity mechanisms, D2 receptors are also expressed in the hippocampus. The firing activity of midbrain dopamine neurons (VTA and SNc) is tightly regulated through an autoinhibitory feedback mechanism in which dopamine release activates D2, GABA​_A_, and GABA​_B_ autoreceptors, opening GIRK channels, hyperpolarizing the neuron, and reducing firing to maintain spontaneous firing patterns. In addition, the activity of midbrain dopamine neurons is suppressed by VTA GABAergic neurons via GABA​_A_ receptor–mediated inhibition (Gangarossa et al. 2012; Olijslagers et al. 2006; Tan et al. 2012; Tsetsenis et al. 2022b).

The anatomical and neurochemical states of VTA innervation of its targets underscore the modulation of the brain centers and refinement of such effects for short and long-term gains. This proposition is supported by previous studies showing that the co-release of dopamine and glutamate in the cognitive centers drives plasticity and plays a critical role in learning, addiction, and developmental neuropsychiatric disorders, including autism, schizophrenia, attention deficit disorders, and obsessive–compulsive disorders (Cai and Tong 2022; Geisler et al. 2007; McNamara and Dupret 2017; Mingote et al. 2019; Ntamati and Luscher 2016; Taylor et al. 2014). Furthermore, disproportionate release or targeted modulation of any of VTA-GABA, dopamine, and glutamate projections leads to changes in learning patterns, valence detection, reward prediction, reward prediction error computation, aversive learning, and reward or drug-seeking behaviors. All of these are linked to aberrant novelty detection systems in which the value of an event is exaggerated or diminished, leading to a dysregulation of adaptive decision-making. Although the neuronal signature of the VTA is mostly dopaminergic, emerging lines of evidence suggest that minority VTA cell populations, such as glutamatergic and GABAergic neurons, disproportionately affect circuit dynamics relative to their estimated sizes and project to brain regions similar (such as nucleus accumbens) to VTA dopaminergic neuronal projections (Beier et al. 2015; Morales and Margolis 2017). For instance, VTA glutamatergic neurons exert a strong excitatory control on forebrain targets, driving reinforcement even without co-release or co-transmission of VTA dopamine. In addition, vesicular transport of glutamate in VTA neurons promotes the storage of dopamine and its co-release with glutamate (Hnasko et al. 2010). Moreover, VTA GABAergic neurons can inhibit dopaminergic output from the VTA, thereby suppressing consummatory behaviors related to reward (van Zessen et al. 2012). Although numerical abundance may be important for determining relative effects or importance, this might not always be the case, especially in the VTA, where non-dopaminergic neurons, such as glutamatergic and GABAergic neurons, have been shown to exert a strong influence even in the absence of dopamine (Zell et al. 2020; van Zessen et al. 2012; Morales and Margolis 2017). As such, numerical abundance alone may not always predict the proportionate degree of causal influence on downstream circuits.

**Fig. 1:**
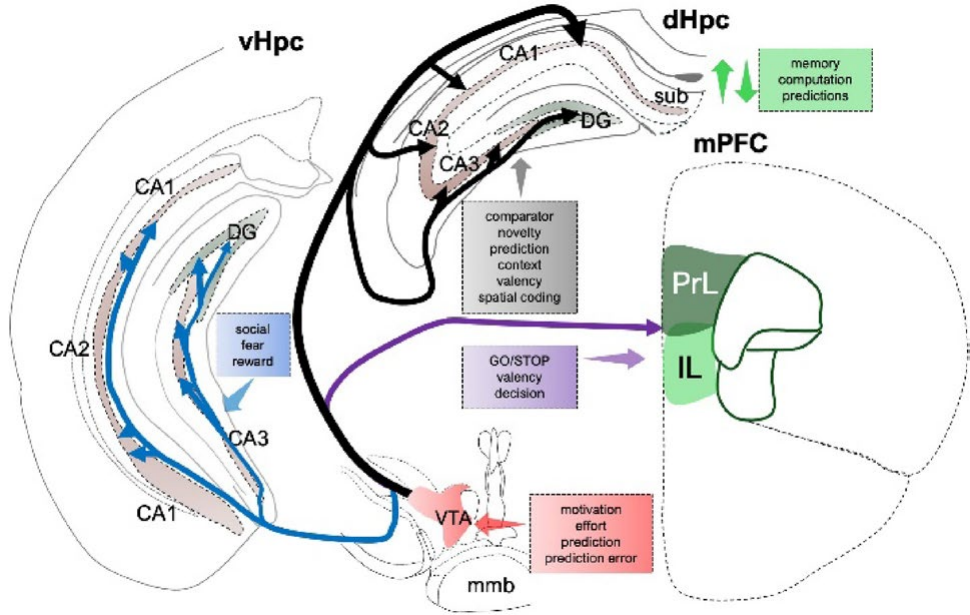
Schematic illustration of VTA projections to the hippocampus and medial prefrontal cortex layers. Discrete combinations of VTA dopamine, glutamate, and GABA presynaptic axon innervates the dorsal–ventral hippocampus and medial prefrontal cortex. These axons are involved in hippocampal computation of novelties and cortical detection of valence for experience-guided executive decisions. These two VTA tracts (“NOVELTY” and “GO-STOP”) functionally overlap in cognitive processes that underlie adaptive behaviors

This review evaluates the functional continuum between the VTA projections that innervate the hippocampus and those that innervate the prefrontal cortex (infralimbic and prelimbic) to elucidate their roles in novelty detection and guided decisions involving reward gradient discrimination, aversion, and risk-taking behaviors. Here, we explore the roles of VTA-dopaminergic, glutamatergic, and GABAergic neuronal projections to the cognitive centers to ascertain their roles in novelty detection, context discrimination, reward-based judgment, and risk assessment (Fig. 1). Furthermore, we enumerate how the VTA-forebrain loop is implicated in several neuropsychological and neurodevelopmental disorders, such as Alzheimer’s disease, Parkinson’s disease, autism, and schizophrenia.

## Anatomical overview of the ventral tegmental area (VTA)

The VTA is a midbrain structure with close lateral association with the substantia nigra, and medial associations with the cerebral aqueduct and fourth ventricle. It is bordered dorsally by the red nucleus and ventrally by the mammillary body of the hypothalamus (Trutti et al. 2021). The VTA is important for reward/aversion-related behaviors, learning, and memory (Ntamati and Luscher 2016; Cai and Tong 2022). Specifically, behavioral and electrophysiological investigations suggest that the VTA is the brain structure responsible for encoding and acquiring rewarding and aversive information (Beier et al. 2015; Bouarab et al. 2019; Castillo Diaz et al. 2022; Cowan et al. 2021; Han et al. 2020; Montardy et al. 2019; Nguyen et al. 2021; Ntamati and Luscher 2016; Tang et al. 2020; Zell et al. 2020). After encoding rewarding or aversive information, the VTA transmits this information to the cognitive centers to promulgate goal-directed behaviors (Gruber and McDonald 2012; Rinaldi and Lefebvre 2016; Lisman and Grace 2005).

**Fig. 2:**
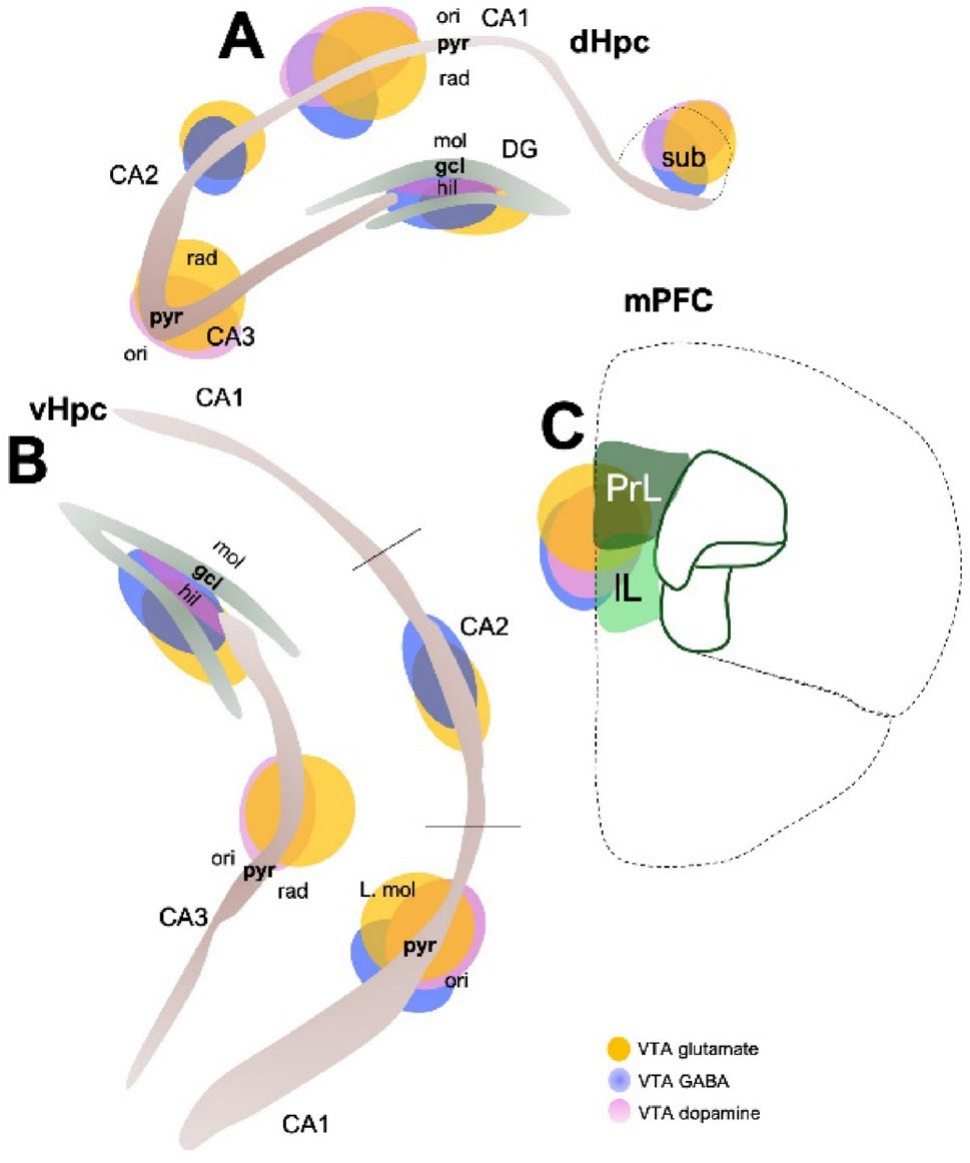
Schematic illustration of layer or region-specific distribution VTA terminals in the hippocampus and prefrontal cortex. Differential distribution of VTA dopamine, glutamate, and GABA terminals in the regions or layers of the **A** dorsal hippocampus **B** ventral hippocampus, and **C** medial prefrontal cortex

The VTA is composed of a heterogeneous population of dopaminergic (60–65%), GABAergic (35%), and glutamatergic (2–5%) neurons (Bouarab et al. 2019; Cai and Tong 2022; Ntamati and Luscher 2016). In addition to dopamine, GABA, and glutamate, the VTA releases other neuromodulatory molecules such as cholecystokinin, neurotensin, corticotropin-releasing factor, brain-derived neurotrophic factor, and calbindin (Cai and Tong 2022). Along with its heterogeneous cellular population, the VTA is also heterogeneous in its afferent and efferent connectivity (Morales and Margolis 2017). The VTA makes direct synaptic contacts with multiple brain regions, including the prefrontal cortex (PFC) (Fig. 2C), nucleus accumbens, pedunculopontine tegmentum, laterodorsal tegmentum nucleus, lateral habenula, periaqueductal gray, bed nucleus of the stria terminalis, lateral hypothalamus, ventral palladium, dorsal raphe nucleus, and dorsal and ventral hippocampus (Fig. 2A, 2B) (Cai and Tong 2022; Kramar et al. 2021; Edelmann and Lessmann 2018).

The role of dopamine in and from the VTA is well-studied and is known to mediate motivated behaviors (Keleta and Martinez 2012). Different subpopulations of dopaminergic neurons are associated with distinct neural networks; therefore, each subpopulation potentially mediates the diverse functions of the VTA (Morales and Margolis 2017). The role of GABA in the VTA is closely related to dopamine, as GABA neurons are the primary source of inhibitory tone to the dopaminergic neurons (Cai and Tong 2022). Thus, VTA GABA neurons play an essential role in behavioral regulation since they form synapses with VTA resident dopamine neurons and other non-dopaminergic neurons, such as glutamatergic neurons in downstream VTA targets like the accumbens (Cai and Tong 2022; Bouarab et al. 2019). Specifically, GABAergic neurons in the VTA have been shown to modulate reward acquisition, stress, and sleep by altering dopamine release (Cai and Tong 2022). Despite the limited studies on the glutamatergic system in the VTA, recent studies suggest that glutamate from the VTA regulates reward reinforcement, aversive behaviors, wakefulness, and defensive behaviors (Cai and Tong 2022). Furthermore, there is evidence that glutamate is also a regulator of dopaminergic neurons in the VTA (Yu et al. 2019; McGovern et al. 2023; Descarries et al. 2008; Morales and Margolis 2017; Root et al. 2020). While dopamine from the midbrain substantia nigra pars compacta (SNc) is important for motor control, VTA dopamine regulates decision-making, reward, motivation, and working memory through its projections to the nucleus accumbens (NAc) and the prefrontal cortex (PFC). Additionally, VTA dopamine is crucial for anticipatory behaviors during reward seeking (Hou et al. 2024; Hughes et al. 2020). Similar to dopaminergic projections from the VTA, both GABAergic and glutamatergic neurons project to distal brain regions dependently and independently of the dopaminergic projections and regulate motivated behaviors in similar fashions (Morales and Margolis 2017).

## The hippocampus integrates diverse presynaptic inputs and has clinical implications.

The hippocampus is a structure that sits deep within the temporal lobe and forms a critical part of the limbic system (Anand and Dhikav 2012; Knierim 2015). Its importance is evident from the fact that the hippocampus and many of its connections have been phylogenetically preserved (Brynja Gunnarsdottir and Clare Kelly 2022). Anatomically, the hippocampus is divided into three functional parts (cornus ammonis, CA): CA1, CA2, and CA3 (Fig. 2A, 2B) (Schultz and Engelhardt 2014). Each of these hippocampal regions is anatomically mapped with the pyramidal cell layer containing mostly glutamate neurons—also called principal cells (Fig. 2A, 2B). In addition, the oriens layer contains basal dendrites, while the radiatum layer contains the apical dendrites of the pyramidal neurons. Although the hippocampus mainly contains glutamate neurons, genetic and immunohistochemical labeling indicate three types of interneurons that are positive for parvalbumin (PVB), somatostatin (SST), and cholecystokinin (CCK). These interneurons are also topographically mapped across the layers of the hippocampus (Tzilivaki et al. 2023; Pelkey et al. 2017; Szilagyi et al. 2011; Tukker et al. 2022). PVB neurons are abundant in the pyramidal cell layer (Nitsch et al. 1990; Deng et al. 2019; Yamada et al. 2015), while SST and CCK neurons are found mostly in the oriens and radiatum layers (Chamberland et al. 2024; Rangel Guerrero et al. 2024). The significance of the anatomical organization of the hippocampus—and its associated formation—is demonstrable in ensemble encoding patterns and oscillations. Notably, pyramidal activation of other pyramidal cells and pyramidal-interneuron connections produces population activation and oscillation patterns that propagate information encoding, learning, memory, and synaptic plasticity (Soltesz and Losonczy 2018; Cavalieri et al. 2021; English et al. 2017; Gridchyn et al. 2020; Bocchio et al. 2024). Therefore, disruption of these connections or the resulting brain oscillation patterns has been shown to reduce the propensity for task learning (Gridchyn et al. 2020). In addition, the target postsynaptic hippocampal (formation) cells, including pyramidal cells and interneurons, express a diverse population of receptors that allow the presynaptic inputs originating from other brain regions to moderate excitability, inhibition, long-term plasticity, and network synchrony in the hippocampus (Kullmann and Lamsa 2007; 2011; Lamsa et al. 2007; Lamsa and Lau 2019; Campanac et al. 2013). These diverse presynaptic inputs innervating the hippocampus are vital in neural development, synaptic formation, pruning, and maintenance (Tzilivaki et al. 2023). Within the hippocampus, target receptors for external presynaptic inputs also exhibit differential expression patterns across the dorsal (Fig. 2A) and ventral (Fig. 2B) hippocampus and among layers within each region. These neurochemical and anatomical mapping patterns provide both gross and fine regulation of cellular processes that underscore cognition in the hippocampus. Although single-cell activation patterns can be detected by in vivo recording or imaging techniques, information encoding in the hippocampus is spatiotemporally represented at the ensemble or network level (Unal et al. 2018; Russo et al. 2024).

In the hippocampal formation, excitatory inputs from CA3, dentate granule cells, and long-range axons from the entorhinal cortex drive excitability and long-term potentiation (Hongo et al. 2015; Kimura et al. 2011; Traub and Whittington 2022; Masurkar et al. 2017; Yeckel and Berger 1990). Therefore, the functionality of the hippocampus is a cumulation of local circuits (dentate gyrus to CA3, CA3 to CA1/CA2/CA3, CA1 to CA1, and CA2 to CA1) across its formation (Ishizuka et al. 1990; Cui et al. 2013; Takacs et al. 2012). For example, the dentate gyrus sends major excitatory inputs to the CA3 via mossy fibers (Ishizuka et al. 1990). The pyramidal cells of the CA3 hippocampal layer send Schaffer collaterals to the CA1, recurrent collaterals and associational projections within the CA3, and axonal projections to the CA1 and CA2 (Ishizuka et al. 1990). CA2 pyramidal neurons send strong axonal projections to the CA1, CA2, and CA3 (Cui et al. 2013). Within the CA1, pyramidal cells receive input from CA3 and engage in local recurrent CA1-CA1 connections (Takacs et al. 2012). Together, local circuit dynamics form a core architecture underlying hippocampal computation. The role of the hippocampus as a functional hub is further evident anatomically by the diverse short- and long-range presynaptic inputs from other brain regions. Robust cholinergic, dopaminergic, GABAergic, serotonergic, and adrenergic inputs—among others—have been identified by immunolabeling techniques and genetically driven anterograde co-localization methods (Freund and Antal 1988; Gray 1998; Kramar et al. 2021; Mendez Guerrero et al. 2020; Heneka 2009; Thompson et al. 1993; Nadler 2011). Distinct innervation of the hippocampus by varied presynaptic inputs has also created a functional difference between the dorsal and ventral hippocampus, with the former mainly involved in cognition, spatial memory, and learning, and the latter involved in emotional and stress regulation (Fanselow and Dong 2010; Besnard et al. 2020). For example, dopaminergic innervation to the ventral aspect of the hippocampus is primarily from the medial VTA, regulating fear conditioning and aversive memory consolidation, while the dorsal aspect is scantly furnished by the medial VTA, and largely by the locus coeruleus, mediating the linkage of spatial and contextual memory (Yamasaki and Takeuchi 2017; Kramar et al. 2021; Edelmann and Lessmann 2018; Chowdhury et al. 2022). The locus coeruleus is the chief source of noradrenergic terminals to the hippocampus (Berridge 2008; Seo et al. 2021). The noradrenergic inputs from the locus coeruleus to the hippocampus regulate spatial memory, synaptic plasticity, and neurogenesis (Galgani et al. 2023; Hansen 2017). Additional dopaminergic innervation to the hippocampus is provided by the nucleus accumbens, midbrain raphe nuclei, and substantia nigra pars compacta (Edelmann and Lessmann 2018; Tsetsenis et al. 2021a; Kempadoo et al. 2016). The plethora of presynaptic inputs discussed highlights the hippocampus's role as a comparator of environmental stimuli in the processing of novel and spatiotemporal information. Dysregulation or alteration of these presynaptic inputs to the hippocampus can have significant clinical and behavioral implications.

Dopaminergic projections from the nucleus accumbens and VTA to the hippocampus have been implicated in Alzheimer’s disease and apathetic behaviors in Parkinson’s disease (Cordella et al. 2018; Carriere et al. 2014). Furthermore, noradrenergic terminals from the locus coeruleus are implicated in stress responses, and fear and anxiety-induced learning of aversive behaviors, both independent and dependent of connections to the amygdala (Privitera et al. 2024; Wilson et al. 2024). For instance, local activation of β-adrenergic receptors after noradrenergic release from the locus coeruleus in the dorsal CA1 facilitates associative learning and can ameliorate fear-learning impairments caused by dysregulation of dopamine in the hippocampus (Tsetsenis et al. 2022a). Additionally, the release of noradrenaline from the locus coeruleus mediates the expression of stress-induced genes in the hippocampus (Privitera et al. 2024). The release of norepinephrine by the locus coeruleus is also implicated in focal epilepsy, as well as the memory deficits experienced in Alzheimer’s disease and Down syndrome (Ferraro et al. 1994; Sanchez et al. 2011; James et al. 2021; Szot et al. 2006). The distinct dopaminergic projections from the locus coeruleus and the VTA to the hippocampus have been implicated in Parkinson’s disease and schizophrenia, with under- and over-activation of dopamine, respectively (Grace 2012; Wu and Liu 2024). Cholinergic inputs to the hippocampus from the medial septal area (MSA) are involved in processing aversive stimuli, where deficits in these inputs are linked to decreased social memory (Shivakumar et al. 2025; Seo et al. 2021). Furthermore, decreased activity of MSA cholinergic inputs to the hippocampus is associated with degeneration and memory decline in Alzheimer's disease (Salimi-Nezhad et al. 2023; Heneka 2009). While cholinergic deficits to the hippocampus exacerbate disease processes, cholinergic excess can also negatively affect the hippocampus, leading to impairment in memory consolidation and progression of Alzheimer's disease (Huang et al. 2022). Serotonergic inputs to the hippocampus from the brainstem raphe nuclei distinctly modulate reward and locomotion, with associated dysregulation implicated in chronic stress (Graeff et al. 1996; Hamada et al. 2024; Seo et al. 2021; Chowdhury et al. 2022; Kramar et al. 2021; Mendez Guerrero et al. 2020; Heneka 2009). Moreover, alterations to the dorsal raphe nuclei serotonergic inputs to the hippocampus are implicated in stress response and psychosis in schizophrenia, and inputs to the CA1 are implicated in depressive symptoms and pathogenesis of Alzheimer’s disease (Chen et al. 2024; Kandilakis and Papatheodoropoulos 2025). Disruptions in VTA presynaptic input to the CA1 can lead to behavioral deficits related to autism, schizophrenia, depression, and obsessive–compulsive disorder (OCD), as well as impairment in decision making and learning. A genetic risk of OCD has been linked to excitatory neurotransmission of the hippocampus and cortex, including neurons containing D1- and D2-type dopamine receptors (Strom et al. 2025). Additionally, Alzheimer’s disease is linked to the hyperexcitability caused by the degeneration of VTA dopamine terminals in the hippocampus (Spoleti et al. 2024; Tanzi 2012). Further evidence suggests an association between dysfunctional VTA dopamine neurons and deficits in social behaviors underlying autism spectrum disorder (ASD) (Bariselli et al. 2018). The “dopamine hypothesis”, a longstanding hypothesis associated with schizophrenia, posits that the dysregulation of dopamine underlies the pathophysiology of the disease. Coupled with the fact that most antipsychotic agents are dopamine D2 receptor antagonists (Creese et al. 1976; Seeman et al. 1975) with consistent changes in D2 receptor density observed in schizophrenia patients (Seeman et al. 2006), enhanced dopamine activity within subcortical dopaminergic circuits, including the hippocampus, has also been associated with positive symptoms of schizophrenia (Abi-Dargham 2004; Laruelle and Abi-Dargham 1999). Genome-wide association studies have identified copy number variant (CNV) deletions associated with genes involved in synaptic function and with cytoskeletal protein complexes in schizophrenia, ranking first for statistical significance and effect size, respectively (Marshall et al. 2017; Kirov et al. 2012). In addition, schizophrenia-associated CNVs include deletions in NRXN1, a neurexin-1 gene encoding a synaptic cell-adhesion protein (Marshall et al. 2017). Emerging evidence has also identified excess increased loss-of-function and missense genetic variants in genes associated with synaptic proteins that regulate NMDA receptors, voltage-gated calcium channels, and postsynaptic density in schizophrenia (Singh et al. 2022; Fromer et al. 2014; Purcell et al. 2014; Rees et al. 2019). Significant increases in exome-wide protein-damaging mutations affecting NMDA receptors, AMPA receptors, and synaptic voltage-gated calcium channels have been reported in schizophrenia (Singh et al. 2022). Other genetic mutations, such as the 22q11 deletion, which is associated with an increased risk of schizophrenia, enhance aberrant ex vivo long-term potentiation at CA1-CA3 synapses and cause deficits in hippocampal-linked spatial memory (Earls et al. 2010). Mutations in neuregulin (NRG1), an important gene encoding a neural growth factor, impair ex vivo LTP at CA3-CA1 synapses and have been linked to schizophrenia (Bjarnadottir et al. 2007). Another gene heavily linked to schizophrenia is the ANKS1B gene, which encodes the amyloid precursor protein intracellular domain associated-1 protein (AIDA-1) involved in mediating the composition of synaptic NMDAR subunits (Tindi et al. 2015). Conditional knockout of AIDA-1 attenuates ex vivo LTP at hippocampal CA3-CA1 synapses (Tindi et al. 2015). Together, these genetic variants strongly implicate genes associated with synaptic activity and plasticity in the risk for schizophrenia (Hall et al. 2015; Trubetskoy et al. 2022; Singh et al. 2022). Moreover, a postmortem study using hippocampal tissue from patients with schizophrenia and matched controls found markedly reduced inhibitory synapses in the CA3 and increased excitatory synapses in the CA1 of patients with schizophrenia, indicating an imbalance in excitation and inhibition and consistent with hippocampal hyperactivity (Farmer et al. 2023). Dissecting the fundamental mechanisms driving the critical roles of the VTA-hippocampal loop could provide insights into its involvement in psychological and neurodevelopmental disorders, aiding in the development of new therapeutics.

## VTA-hippocampus loop and novelty detection

VTA projections to the hippocampus consist of dopamine, glutamate, and GABA axon terminals, with varying distribution across the hippocampal regions and layers (Soden et al. 2020; Sanchez-Catalan et al. 2014; Cai and Tong 2022; Ghanbarian and Motamedi 2013). In addition to the VTA, the substantia nigra pars compacta (SNc) also sends direct dopaminergic projections to the dorsal hippocampus, particularly the CA1 region. Together, the VTA and SNc contribute approximately 15–18% of the dopaminergic terminals within the hippocampus. This midbrain-derived dopamine is critical for contextual fear memory and supporting the formation of aversive memories through hippocampal ensembles (Gasbarri et al. 1994; Tsetsenis et al. 2021b). In addition to dopamine, it is logical that VTA glutamate and GABA inputs are robust in the hippocampus since glutamate and GABA neurons are hippocampal resident cells and express an abundance of compatible receptors. The functional significance of VTA-driven modulation of the hippocampus by co-releasing dopamine, GABA, and glutamate combinations relative to learning or behavioral events is still poorly understood to date. However, recent experiments have attempted to fill in some of these gaps using pharmacology, optogenetics, and chemogenetics to modulate one or more VTA inputs concurrently during reward or aversion tasks.

In describing the VTA-hippocampus loop, VTA projections to the functionally distinct dorsal, intermediate, and ventral hippocampus ought to be considered (Fanselow and Dong 2010; Jarzebowski et al. 2022; Olsen et al. 2023; Takita et al. 2013). Putative dorsal CA1 neurons encode spatiotemporal events related to location and contexts. These place cells increase their firing rate around specific locations called “place fields”. The size of place fields and peak firing rate have been shown to be moderated by contextual changes involving reward and aversion. In addition to place cells, recent studies showed that specialized cells within the dorsal CA1 (dCA1) are “reward cells” (Moita et al. 2003; Gauthier and Tank 2018). These neuron types increase their firing rate at locations where a reward is present and change the peak firing position to align with shifting reward locations (Asgeirsdottir et al. 2020; Blair et al. 2023; Czurko et al. 2011; Geiller et al. 2017; Jarzebowski et al. 2022; Knierim et al. 2006; Robinson et al. 2020; Xiao et al. 2020). Since reward locations are equally encoded by place and reward cells, their overlap and significance in reward learning are now being examined at the network level (Gauthier and Tank 2018; Jarzebowski et al. 2022; Robinson et al. 2020; Xiao et al. 2020). Thus, it is likely that the cumulation of spatiotemporal activity of these different ensembles (*reward, place, and context groups*) of cells within the larger dCA1 population encodes information about space, contexts, and rewards. The role of the VTA in the functionality of the dCA1 ensembles cannot be overemphasized. It follows that modulation of VTA dopamine, glutamate, and GABA inputs alters the size and precision of information coding of dCA1 cells (Martig and Mizumori 2011b; Wirtshafter and Wilson 2020).

The effect of VTA projections on the hippocampus is also linked to the topographic distribution of its diverse terminals along the rostro-caudal and dorsoventral axis of the hippocampus. The intermediate hippocampus—less studied than *the dCA1 and vCA1*—has been shown to be different in its encoding pattern of space and goal-oriented behaviors compared to the dorsal hippocampus (Jarzebowski et al. 2022). Furthermore, the ventral hippocampus, which is more in tune with social interactions and adaptive behavior, has more VTA innervation than the dorsal hippocampus (Hong and Kaang 2022; Okuyama et al. 2016). This is logical given that the vCA1 regulates fear conditioning and associated behavioral expression through its monosynaptic infralimbic (IL) cortical inputs (Brockway et al. 2023; Wang et al. 2018). The functional diversity of the hippocampus based on its topographical gradient further defines the complexity of the VTA-hippocampus loop, such that VTA modulation of the hippocampus in anteroposterior dimensions can produce divergent results ranging from spatial learning to reward context discrimination and aversion.

The VTA also receives long-range glutamatergic inputs from the dorsal, intermediate, and ventral hippocampus. The functional significance of this innervation gradient is still poorly understood. Nonetheless, there is robust evidence that hippocampal-linked events—such as novelty—increase VTA dopamine release, an effect similar to VTA activation patterns during reward encounters (Engelhard et al. 2019; Fleury et al. 2024; Martig and Mizumori 2011b; 2011a). A key function of the bidirectional VTA-hippocampus circuit is the detection of novelty. This novelty detection is critical for adaptive behaviors and is associated with plasticity-related changes within the hippocampus. Evidence suggests that novelty signals are transmitted through a polysynaptic pathway that drives novelty-dependent activation of dopaminergic neurons in the VTA (Lisman and Grace 2005). Interestingly, this response in the VTA occurs even in the absence of reward, indicating that novelty itself serves as a potent signal arising from the hippocampus. This raises the possibility that novelty-evoked dopamine release may influence behavior in ways similar to reward-evoked dopamine. In addition, the hippocampal representation of new information not only modulates VTA dopamine neurons but also impacts the substantia nigra, and this broader loop may facilitate ongoing interactions with cortical circuits (Baldassarre et al. 2013; Cowan et al. 2021; Lisman and Grace 2005). It follows that the complex computation of environmental neural maps between the hippocampus and VTA is important for learning reward locations and subsequently predicting or generating efforts to acquire rewards at those locations (Davidow et al. 2016; Gomperts et al. 2015; Liu et al. 2023; Stetsenko and Koos 2023). Moreover, the functions of VTA dopamine and GABA neurons in reward prediction and reward prediction error are well documented (Kaushik et al. 2022; Lammel et al. 2014; Mohebi et al. 2019; Solie et al. 2022; Takahashi et al. 2016). Despite the progress, several important underpinnings of these computations remain elusive. Specifically, the ensemble significance of dorsal *versus* ventral hippocampal activation in VTA events remains unclear in several behavioral paradigms involving spatial coding and adaptive reward or aversive responses. It is possible that a dichotomy occurs, with dorsal CA1 activation of the VTA driving space contextualization, while ventral CA1 activation of the VTA provides context about social encounters or adaptive events. Additionally, there are other types of non-dopaminergic neurons in the VTA whose function and role in the overall computation of VTA-linked behaviors are relatively unknown, and the investigation of the physiology of those cells has just recently gained momentum in the broader field (Breton et al. 2019; Cai and Tong 2022; Oriol et al. 2024).

Disruptions in VTA presynaptic input to the CA1 can lead to behavioral deficits related to autism, schizophrenia, depression, and obsessive–compulsive disorder (OCD), as well as impairment in decision making and learning. A genetic risk of OCD has been linked to excitatory neurotransmission of the hippocampus and cortex, including neurons containing D1- and D2-type dopamine receptors (Strom et al. 2025). Additionally, Alzheimer’s disease is linked to the hyperexcitability caused by the degeneration of VTA dopamine terminals in the hippocampus (Spoleti et al. 2024; Tanzi 2012). Specifically, degeneration of VTA dopaminergic neurons reduces hippocampal dopaminergic innervation that impairs (firing activity) gamma-waves driven by parvalbumin interneurons, diminishes inhibition of pyramidal neurons, and ultimately induces hippocampal excitability (Spoleti et al. 2024; Tanzi 2012). Further evidence suggests an association between dysfunctional VTA dopamine neurons and deficits in social behaviors underlying autism spectrum disorder (ASD) (Bariselli et al. 2018). Dissecting the fundamental mechanisms driving the critical roles of the VTA-hippocampal loop could provide insights into its involvement in psychological and neurodevelopmental disorders, aiding in the development of new therapeutics. Imaging and postmortem studies consistently reveal altered hippocampal structure and function in schizophrenia patients (Tamminga et al. 2010). Particularly, during memory recall tasks in imaging studies, these patients consistently fail to recruit hippocampal ensembles (Heckers et al. 1998; Weiss et al. 2003). Moreover, attenuated hippocampal volume in the brains of schizophrenia patients has been a consistent anatomical finding (Harrison 1999; Nelson et al. 1998). In an animal model of schizophrenia, Lodge and Grace (2007) demonstrated that an enhanced drive from the hippocampus can cause hyperexcitability of the VTA specifically by increasing the spontaneous activity of VTA dopaminergic neurons (Lodge and Grace 2007). This link was completely reversed following hippocampal inactivation, showing a direct link between hippocampal dysfunction and hyperactivity of VTA dopaminergic neurons that underlie psychosis in schizophrenia (Lodge and Grace 2007). In a resting-state functional magnetic resonance imaging (rsfMRI) study, functional coupling between VTA-hippocampus was significantly increased in individuals with first episode psychosis compared to healthy controls (Gregory et al. 2021). Additionally, there was a significant correlation between this high VTA-hippocampal functional coupling and individual variation in psychosis-related symptomology (Gregory et al. 2021). Similarly, another rsfMRI study revealed reduced VTA-hippocampal connectivity in children and adolescents exposed to early life stressors (Marusak et al. 2017). Reduced signaling in the VTA has been observed in patients with psychosis in response to various forms of salience, including novelty and negative emotions, during a visual oddball task (Knolle et al. 2018). Emerging lines of evidence also show that inactivation of the VTA by dopamine antagonists can attenuate the occurrence of behavioral patterns and symptomology associated with post-traumatic stress disorder (PTSD) (Corral-Frias et al. 2013).

### Integration of novelty and adaptive behaviors in the VTA-hippocampus loop

The hippocampus creates a neural map of the environment. Since environmental inputs are continuous, the neural map must be continuously updated through novelty detection. The “continuous update hypothesis” posits that hippocampal representations and neural maps are dynamic and continuously modified or updated to integrate new experiences (novelty) and responses to salient events. Therefore, when rewards are presented or following experimental modulation of the VTA, place field size and position are altered (McNamara et al. 2014; Krishnan et al. 2022; Sosa and Giocomo 2021). Similarly, network-level adjustments have been described in hippocampal ensembles when aversive events are presented or predicted (Okada et al. 2017; Fyhn et al. 2002; Nyberg et al. 2022). These concepts support the continuous update hypothesis, the idea that the hippocampus receives, integrates, and continuously maps environmental stimuli relative to VTA-dependent events.

VTA-hippocampal loop embodies the integration of novelty detection with experience-based learning to guide future decisions (Lisman and Grace 2005; Ripolles et al. 2016). Given that the hippocampus compares previous memories to newly acquired or updated neural information about the environment, it is logical to explore recent advances in the neural circuit mechanisms for valence detection and context discrimination. Although the VTA is emphasized, other brain regions in the mesocorticolimbic system play direct roles in the overall computation. The CA1 serves as a comparator that receives novel sensory cortical inputs through the long-range entorhinal cortex (Bell et al. 2021; Knierim et al. 2006; Masurkar et al. 2017; Stepan et al. 2015; Takacs et al. 2012; Traub and Whittington 2022; Tukker et al. 2022) and short-range CA3 projections (Hongo et al. 2015; Lisman 1999; Martig and Mizumori 2011b; Oh et al. 2016; Soltesz and Deschenes 1993). This information received by the CA1 is then dispersed onto thousands of spiny nucleus accumbens neurons that compute saliency in conjunction with PFC accumbens inputs. Through the combined input from the hippocampus (regarding novelty) and the PFC (weight assignment and memory storage priority), the nucleus accumbens can process the latest information necessary for further transmission. Activation of the nucleus accumbens inhibits the ventral pallidum through its GABA projections (Keleta and Martinez 2012). Since the ventral pallidum sends an inhibitory signal to the VTA through its GABA projection (Keleta and Martinez 2012), accumbens suppression of pallidum activity results in a disinhibitory pathway for VTA activation. The VTA also receives excitatory glutamatergic and cholinergic inputs from the pedunculopontine nucleus of the amygdala (Lisman and Grace 2005). These inputs supplement the role of the PFC, as they provide additional information about the presence of salient stimuli (Lisman and Grace 2005).

VTA dopaminergic neurons are activated through the combined activation of the VTA by the disinhibitory pathway of the accumbens and excitatory amygdala and brainstem cholinergic inputs. There is anatomical and electrophysiological evidence that the VTA dopamine-releasing terminals innervate hippocampal pyramidal cells and significantly regulate memory encoding and synaptic long-term potentiation (Keleta and Martinez 2012; Lisman and Grace 2005). In summary, the VTA-hippocampus loop starts when the hippocampus detects newly acquired information not stored in its long-term memory. Newly arrived information, along with other sources of information that detect saliency, activates the VTA. After processing the novelty and saliency of information, the VTA activates its projections to the hippocampus. Although predominantly thought to be dopaminergic, there is now evidence that VTA glutamate inputs to the hippocampus and other forebrain centers are robust and can regulate network synchrony and synaptic plasticity (Adeniyi et al. 2020; Adeyelu and Ogundele 2023; Han et al. 2020; Montardy et al. 2019; Ntamati and Luscher 2016; Perez-Lopez et al. 2018; Root et al. 2014; Shrestha et al. 2020; Yoo et al. 2016; Zell et al. 2020). With evidence that VTA dopamine and glutamate are co-released in the hippocampus, it is necessary to determine how the dopamine/glutamate balance is achieved and what their alterations mean for behavioral events dependent on the loop.

There is strong support for this classical understanding of the VTA-hippocampus loop. Optogenetic enhancement of the dopaminergic VTA projections in the dorsal hippocampus improved single-day learning in rodents (McNamara and Dupret 2017). It was also found that the binding of dopamine in the D1/D5 receptors in the hippocampus promotes attention, episodic memory formation, and spatial learning (Kempadoo et al. 2016). In an fMRI human study, learning tasks involving explicit and implicit rewards activated the dopaminergic midbrain, including the VTA, hippocampus, and ventral striatum. While the support for the classical understanding is strong, there is more nuance to the VTA-hippocampus loop than once thought. Although the VTA-hippocampus loop is thought to be primarily dopaminergic, robust GABA and glutamate VTA projections have also been demonstrated in the hippocampus (Ntamati and Luscher 2016; Cai and Tong 2022; Geisler and Wise 2008; Gorelova et al. 2012; Han et al. 2020; Lisman and Grace 2005). Thus, knowing that motivational signals from the VTA can arise from non-dopaminergic neurons, there are still gaps in the overall understanding of non-dopaminergic VTA projections and their significance within the loop (Morales and Margolis 2017; Yoo et al. 2016).

While the research on the dopaminergic basis of learning has focused on the VTA-hippocampus loop, the fiber density of dopaminergic neurons from the locus coeruleus to the hippocampus is 4.5 times greater than the fiber density of the dopaminergic neurons from the VTA to the hippocampus (McNamara and Dupret 2017; Takeuchi et al. 2016). The locus coeruleus (LC) is known primarily for releasing norepinephrine (NE) alongside dopamine. However, recent studies using advanced genetic tracing and electrophysiology have demonstrated that the LC can co-release dopamine (DA) and NE specifically in the dorsal hippocampus (dCA1), contributing to novelty detection and spatial memory formation. Interestingly, NE can independently act on D2 receptors to activate dopamine-related signaling pathways, whereas dopamine primarily engages D1 receptors to facilitate spatial learning and long-term potentiation (LTP). Additionally, LC-derived dopamine contributes to novelty-related responses, a feature not typically mediated by the ventral tegmental area (VTA) (Takeuchi et al. 2016; Tsetsenis et al. 2022b). This does not discount the relationship between VTA dopaminergic projections to the hippocampus and learning. However, it suggests that the VTA-hippocampus loop is not the only or primary source of dopaminergic-based learning. Optogenetic modulation revealed that, while dopaminergic neurons in the VTA respond to novelty, they do not influence the "novelty effect", where memory encoding is enhanced after a learning period when novel stimuli are introduced (Takeuchi et al. 2016). Conversely, enhancement of the dopaminergic signals from the locus coeruleus increased the ability of rodents to use the “novelty effect” (Takeuchi et al. 2016). This result can be partially understood based on the evidence that the dopaminergic innervation from the VTA is mainly in the ventral region of the hippocampus, which is more associated with learned fear and anxiety (Kempadoo et al. 2016).

Aside from the VTA-hippocampus loop being an essential pathway for memory and learning, it is also implicated in a variety of neurological and psychiatric disorders, including addiction/substance use disorders, schizophrenia, Alzheimer’s, and mood disorders (Anand and Dhikav 2012). The plastic nature of the hippocampus makes it vulnerable, partly accounting for these associated disorders (Schultz and Engelhardt 2014). Additionally, changes to the dopaminergic system through drug abuse, chronic stress-inducing changes in the GABAergic system, and a hyperdopaminergic state interfering with novelty detection in schizophrenia are all ways that a dysfunction in the VTA-hippocampus loop can lead to pathologies (Keleta and Martinez 2012; Lisman and Grace 2005; Cai and Tong 2022; Heysieattalab et al. 2016). Owing to the importance of the dopaminergic system in the VTA-hippocampus loop, dopamine in the VTA has been a primary therapeutic target for treating disorders related to drug addiction and mood disorders (Cai and Tong 2022).

## Role of VTA neurons in cortical decision-making

A key part of learning, through novelty detection, is to rank environmental and contextual inputs by valence determination. This process is computed through neural pathways and ensembles in the hippocampus, amygdala, medial prefrontal cortex, and nucleus accumbens (Bayer and Bertoglio 2020; Breton et al. 2019; Brockway et al. 2023; Capuzzo and Floresco 2020; Chen et al. 2021; de et al. 2023; Green and Bouton 2021; Hefner et al. 2008; Hoover and Vertes 2007; Johnson et al. 2021; Kim and Han 2016; Moorman and Aston-Jones 2015; Nett and LaLumiere 2021; Riaz et al. 2019; Shipman et al. 2018; Wang et al. 2018). Assigning value to novel information is key to determining what is stored in long-term memory, thereby preventing memory overload. Thus, based on this value system, information can be retrieved later for comparison with newer or updated neural maps for continuous novelty and learned experience. This cognitive process is pertinent for guided decision-making, where past experiences are applied to make decisions that benefit the animal, a key part of neural adaptations (Fig. 1). In general terms, the neural circuitry for novelty detection and those associated with decision-making guide behavioral expression that anticipates reward and avoids punishment. Several behavioral tasks have been used to study these circuits. Most notably, aversion-driven memory traces are strong, and their circuits are well documented in decision-making. Conditioned (CS) and unconditioned (US) aversive stimuli such as foot shock, social defeat, conditioned place aversion, and excessive light or sounds have been used to read out the function of these circuits (Glover et al. 2023; Vander Weele et al. 2018; Yan et al. 2019). Whereas reward tasks assess similar circuits to determine their role in motivation, reward seeking behaviors, and place preference (Bals-Kubik et al. 1993; Otis et al. 2017; Granon and Changeux 2012).

The integration site of inputs that connotes decision has been mapped primarily to the prefrontal cortex, a brain region for executive cognitive function. With its three anatomically distinct regions, the prelimbic (PrL) and infralimbic (IL) areas have taken center stage through dichotomous roles in reward and aversive learning (Bayer and Bertoglio 2020; Brockway et al. 2023; Capuzzo and Floresco 2020; Chen et al. 2021; Green and Bouton 2021; Johnson et al. 2021; Moorman and Aston-Jones 2015; Nett and LaLumiere 2021; Riaz et al. 2019; Shipman et al. 2018; Wang et al. 2018). Although generally expressed as PrL (GO)/IL (STOP) concepts, recent studies of pyramidal cell populations in the PrL and IL showed that the directionality of neuronal firing during behavioral events is broader than usually expected. As such, it is now evident that population dynamics, rather than individual neuronal activity, provide an adequate representation of reward and aversion encoding in these brain areas (Moorman and Aston-Jones 2015). With some neurons showing an increase, decrease, or no change in firing rate patterns, the net firing rate is mainly determined by the group of neurons with the predominant directionality. Neuronal firing patterns have been shown to impact learning, as continuous tonic stimulation of VTA-PFC dopaminergic projections preserves cue-reward associations, and phasic stimulation causes deviation from learned rewards (Ellwood et al. 2017).

Both the PrL and IL project to and are innervated by the VTA (Chiba et al. 2001). Projections from the IL and PrL to the VTA (Fig. 2C) have been shown to have distinct and opposing roles in VTA firing, with PrL suppression decreasing the firing of VTA dopaminergic neurons and IL inactivation increasing VTA dopaminergic firing (Patton et al. 2013). Furthermore, VTA dopamine and glutamate neurons send long-range inputs to the medial PFC (mPFC) and indirect inputs through the accumbens. Therefore, it is logical that network activation of VTA and cortical neurons is pertinent for decision-making. In support of this proposition, both the VTA and these cortical regions are heavily linked to the ventral CA1, a brain region involved in adaptive learning. Recent studies showed that the timing of IL/PrL activation, relative to VTA dopaminergic and non-dopaminergic neuronal activation, underscores behavioral expression related to reward or aversive learning. This has been extensively studied in absolute tasks with high probabilities for acquiring a reward or encountering an aversive stimulus (Capuzzo and Floresco 2020; Coley et al. 2021; Dalenberg et al. 2018; Han et al. 2020; Redondo et al. 2014). Additionally, the connection between medial cortical neurons and the VTA has been associated with risk tolerance, impulsivity, and addiction behaviors (Muñoz-Villegas et al. 2017). Activation of the mPFC to VTA pathway in rats with low avoidance behaviors was shown to decrease impulsivity, while inhibition of this pathway drives the opposite effect (Urueña-Méndez et al. 2024).

Although the concepts described here are pertinent for understanding neural circuit control of behavior and decision, there is a limitation to real-life translation since contingencies in an animal’s habitat will usually present both rewards and risks. In the past decade, this area of research has taken off, with several studies now introducing reward/risk paradigms to assess the neural computation of risk when a reward acquisition event is paired with the risk of an aversive stimulus (Lammel et al. 2012). Several experiments employing ex vivo* and *in vivo ensemble recording or imaging methods have been used to better understand the computation of reward/risk decisions across neural circuits (Park and Moghaddam 2017; Lammel et al. 2012). Recent evidence now shows that the functional dichotomy, as initially thought, is not all or none. Instead, based on inputs from the hippocampus, VTA, amygdala, and other regions, the directionality of the cortical firing rate is tuned to represent a state and continuum between states.

### Role of the VTA in reward-guided decisions

Dopamine neurons are the most abundant in the VTA, accounting for more than 60% of the characterized population. Previous studies have shown that VTA dopamine affects action sequence performance when contingencies are introduced to alter reward retrieval and reward-seeking behaviors (Halbout et al. 2019). Evidently, the firing rate of dopaminergic and non-dopaminergic neurons is moderated to reflect unexpected rewards, correct reward predictions, and unexpected reward omissions (Keiflin and Janak 2015; Nasser et al. 2017). All of these have been examined vigorously to decode the role of VTA neuron ensembles in correctly predicting these events and to understand how the ensembles behave when the prediction leads to an unexpected result.

To assess the role of dopamine on reward-driven decision-making, a study of 31 healthy subjects was conducted. A subgroup received (i) a dopamine-boosting drug (L-DOPA), (ii) a dopamine blocker (amisulpride), and (iii) a placebo. The results showed that L-DOPA increases dopamine levels, which impacts decisions based on the likelihood and size of the reward. Conversely, amisulpride—a dopamine 2 receptor (D2R) antagonist—decreased the impact of reward size and probability of acquiring a reward (Antonia Dias Maile et al. 2024). Other studies have also elucidated the complex coding patterns that VTA dopaminergic and GABAergic neurons employ during reward-related tasks. There is substantial evidence in human fMRI and rodent studies that VTA neurons encode mental representations to support judgments dependent on short-term memory (Glykos and Fujisawa 2024). This result was further corroborated by a study that combined VTA dopamine recording with exogenous modulation of dopamine signals during decision-making behavioral tasks (Stopper et al. 2014). Their result showed that dopamine facilitates learning from past reward experiences, thus increasing the accuracy of decisions for the reward acquisition task (Stopper et al. 2014). In addition, reward-guided activities impact the circadian clock of VTA dopamine, as indicated by diurnal food choice decisions. It also changes the presynaptic release of dopamine from the VTA terminals at their target site in the accumbens (Koch et al. 2020). Another study reported that the chemogenetic inhibition of VTA dopaminergic neurons decreases the motivation associated with seeking reward-related cues without affecting the motivation to seek reward (Halbout et al. 2019).

### Risky decision-making and risk assessment

Several studies in the past decade have emphasized research areas that consider the reward-aversion continuum, particularly with cued and uncued stimuli associated with rewards and the risk of punishment. Park and Moghaddam recently highlighted the advancement in the study of this neural pathway or circuitry (Park and Moghaddam 2017; Lammel et al. 2012). They hypothesized that neurons involved in action driven by reward are also connected to the risk of punishment. Their results further demonstrate that the neural network between VTA and mPFC assesses the risk of punishment during reward-guided actions (Park and Moghaddam 2017). The direct link between VTA dopamine release and risk-aversion behavioral expressions is further supported by the work of Stopper and colleagues (Stopper et al. 2014). As such, enhanced dopaminergic activity in the VTA increased risk tolerance. Thus, in experiments where animals received foot shock as a risk in a reward-driven task, rats with robust VTA dopamine release appeared to have a higher risk tolerance (Lichtenberg et al. 2018; Park and Moghaddam 2017). Furthermore, a high dopamine response was associated with risky decision-making processes (Freels et al. 2020). Finally, the regulation of risk assessment and moderation of risky decision-making tendencies by VTA neurons, especially dopamine, seems to be nearly unanimous amongst all related research.

## Conclusion

The VTA-hippocampus loop is pivotal in the complex process of learning and memory, with dopamine, GABA, and glutamate from the VTA facilitating various cognitive functions. By detecting new information and working in tandem with salient information from the PFC and amygdala, the hippocampus activates the VTA to facilitate plasticity, leading to memory encoding. Although research has predominantly focused on the dopaminergic aspect of the VTA-hippocampus loop, recent findings emphasize the potential significance of the GABAergic and glutamatergic projections in motivated behaviors and learning. The VTA-hippocampus loop and VTA-PFC axis are functional continua such that novelties detected in the loop are computed to refine decisions guided through the VTA-PFC axis. Therefore, a holistic understanding of the role of the VTA in decision-making is contingent on the integration of novelty detection and context discrimination regimes across the forebrain cognitive centers.

## Data Availability

No datasets were generated or analysed during the current study.
